# Genome-Wide Identification and Evolutionary Analysis of Argonaute Genes in Hexaploid Bread Wheat

**DOI:** 10.1155/2021/9983858

**Published:** 2021-06-18

**Authors:** Yan-Feng Liu, Li-Min Wang, Li-Zi Zhao, Wei Wang, Hong-Xia Zhang

**Affiliations:** ^1^The Engineering Research Institute of Agriculture and Forestry, Ludong University, 186 Hongqizhong Road, Yantai 264025, China; ^2^School of Resources and Environmental Engineering, Ludong University, 186 Hongqizhong Road, Yantai 264025, China; ^3^School of Life Sciences, Ludong University, 186 Hongqizhong Road, Yantai 264025, China

## Abstract

Argonaute (AGO) proteins play a pivotal role in plant growth and development as the core components of RNA-induced silencing complex (RISC). However, no systematic characterization of *AGO* genes in wheat has been reported to date. In this study, a total number of 69 *TaAGO* genes in the hexaploid bread wheat (*Triticum aestivum* cv. Chinese Spring) genome, divided into 10 subfamilies, were identified. Compared to all wheat genes, *TaAGOs* showed a significantly lower evolutionary rate, which is consistent with their high conservation in eukaryotes. However, the homoeolog retention was remarkably higher than the average, implying the nonredundant biological importance of *TaAGO* genes in bread wheat. Further homoeologous gene expression bias analyses revealed that *TaAGOs* may have undergone neofunctionalization after polyploidization and duplication through the divergent expression of homoeologous gene copies, to provide new opportunities for the generation of adaptive traits. Moreover, quantitative real-time polymerase chain reaction (qRT-PCR) analyses indicated that *TaAGO* gene expression was involved in response to heat, drought, and salt stresses. Our results would provide a theoretical basis for future studies on the biological functions of *TaAGO* genes in wheat and other gramineous species.

## 1. Introduction

Small RNA- (sRNA-) guided gene silencing is crucial for the maintenance of genome stability and plant response to environmental alterations. Argonaute (AGO) family proteins function as the major components in this RNA silencing machinery. They associate with sRNAs such as short interfering RNAs (siRNA) and microRNAs (miRNAs), to form RNA-induced silencing complexes (RISC), which induce sequence-specific degradation or repress the translation of mRNAs [1]. AGO family proteins are highly conserved in eukaryotes, containing several typical domains such as the N-terminal, Piwi Argonaute Zwille (PAZ), MID, and PIWI domains. The N-terminal domain participates in the loading and unwinding of small RNA duplexes, whereas the PAZ and MID domains anchor the 3′- and 5′-ends of the sRNA, respectively. The PIWI domain is structurally similar to RNaseH and is responsible for the cleavage of its target mRNA.

In Arabidopsis, a total number of 10 AGO proteins, which are divided into three phylogenetic clades: AGO1/AGO5/AGO10, AGO2/AGO3/AGO7, and AGO4/AGO6/AGO8/AGO9, have been detected. *AGO1* regulates plant development through the miRNA-mediated pathway. A loss of function in *AGO1* led to abnormal inflorescence and filamentous structures in the Arabidopsis mutant plants [2]. AGO10 regulates the differentiation of shoot apical meristem (SAM) by sequestering AGO1 from miR165/166, leading to promoted expression of their target homeodomain-leucine zipper (HD-ZIP) proteins [3]. *AGO2* and *AGO3* are similar to each other and may have redundant functions. AGO2 plays a role in antibacterial immunity by binding to miR393b∗, which inhibits the translation of *MEMB12*, a gene repressing the exocytosis of PR1 [4]. No *AGO3* orthologs have been detected in rice and maize [5]. In Arabidopsis, rice, and maize, AGO7 predominantly binds to miR390, which cleaves *TAS3* precursor RNA [6–8]. The cleavage products are converted into double-stranded RNAs by the Suppressor of Gene Silencing 3 (SGS3) and RNA-directed RNA polymerase 6 (RDR6) and then diced by Dicer-like 4 (DCL4) into tasiRNAs, which control the transition from juvenile to adult phase at the vegetative stage [9]. AGO4/6/9 is involved in the *de novo* methylation of CG, CHG (H=A, C, T), and CHH sequence contexts in plants [10]. Mutation in *AGO4* clade genes (*AGO4*, *AGO6*, *AGO8*, or *AGO9*) induces supernumerary female gametophytic precursors in Arabidopsis [11].

In rice, 19 *OsAGOs* have been identified, of which 14 were preferentially expressed at reproductive stage. Collinearity analysis revealed that segmental and tandem duplication contributed to the expansion of AGO family in rice, and duplicated genes have possessed diverged functions during evolution [12]. In pepper, 12 *CaAGOs* were detected. *CaAGO4b*, *CaAGO7*, and *CaAGO10a* were significantly repressed by salt stress, whereas *CaAGO2* and *CaAGO5* were induced by cold stress, suggesting a potential role of *AGOs* in abiotic stress resistance [13]. In *Saccharum spontaneum*, 21 *SsAGOs* were detected based on the genome-wide identification and phylogenetic analyses. Among them, *SsAGO18a* was preferentially expressed in the stem, and *SsAGO2b*, *SsAGO5a*, *SsAGO5c*, *SsAGO6b*, and *SsAGO10c* were significantly induced by osmotic stress [14]. In *Populus trichocarpa*, among the 14 detected *AGOs*, *PtAGO10a*, *PtAGO1b*, and *PtAGO4c* were upregulated in stems, while *PtAGO4b* and *PtAGO5a* were downregulated [15]. In potato, among the 14 identified *AGOs*, *StAGO15* was activated upon pathogen infection [16]. In *Citrus sinensis*, 13 *AGOs* were identified, and the repression of RNA-directed DNA methylation (RdDM) pathway is necessary for fruit abscission [17]. Therefore, AGO proteins could contribute to a diverse set of functions in plants.

Hexaploid bread wheat has been grown as one of the most important cereal crops and provides about 30% of the staple food source for humankind [18]. Abiotic stresses have severely limited global wheat production [19, 20]. sRNA-guided gene silencing at the transcriptional and posttranscriptional levels has been found to have important functions in controlling growth and resistance to biotic and abiotic stresses [21]. To date, several *AGO* genes have been isolated from wheat. *TaAGO1b* and *TaAGO4* were ubiquitously expressed in both vegetative and reproductive organs [22]. *TaAGO5* is involved in the resistance to *Diuraphis noxia* infestation. Decreased expression of *TaAGO5* led to increased susceptibility of wheat *D. noxia* feeding [23]. To further understand the possible functions and the evolutionary dynamic of *AGO* genes in wheat, we performed a systematic analysis on the structural conservatism, evolutionary relationship, and expression pattern of these genes. This work will provide an overview of the AGO family and a new perspective on their possible function and evolution in cereal crops.

## 2. Materials and Methods

### 2.1. Identification and Characterization of Candidate AGO Genes in Bread Wheat

To identify all the candidate *AGO* genes, the bread wheat (*Triticum aestivum* cv. Chinese Spring) high-confidence protein sequences were downloaded from https://urgi.versailles.inra.fr/download/iwgsc/IWGSC_RefSeq_Annotations/v1.0/. The Hidden Markov Model (HMM) profiles of conserved domains of AGO (PF16486, PF02170, and PF02171) were retrieved from the protein family database (Pfam, https://pfam.xfam.org/) and were used as queries to search for *AGO* candidate genes in the bread wheat genome as described previously [16]. All hits with a cut-off value lower than 1*e*-5 were retained and treated as candidate *AGO* genes. The candidate sequences were examined to confirm the existence of the conserved AGO domains using SMART (http://smart.embl-heidelberg.de/), Pfam (http://pfam.xfam.org/), and the Conserved Domain Database (CDD) in NCBI (https://www.ncbi.nlm.nih.gov/). Proteins with anonymous or truncated domains were eliminated to ensure the accuracy of the results. The resultant *AGO* genes were named according to their phylogenetic relationships and subgenome location (A, B, or D). In details, each gene name consists of the abbreviation for *Triticum aestivum* (*Ta*), followed by the subfamily name of rice or maize (e.g., *AGO4a*) and chromosomal location (e.g., 3A). Duplicated genes from the same chromosome were labeled with lower case letters (e.g., a). TBtools, WoLF PSORT (https://wolfpsort.hgc.jp/), ExPASy (http://www.expasy.ch/tools/pi_tool.html), and MCScanX were used to generate exon-intron structures, subcellular localization, isoelectric point, molecular weight, and gene duplication type, respectively [24, 25]. The *AGO* genes in the progenitors, *Triticum urartu* (genome AA), *Aegilops tauschii* (genome DD), and *Triticum turgidum* L. ssp. *durum* (genome AABB), were detected using the same methods.

### 2.2. Evolutionary Rate Calculation of Bread Wheat Genes

The evolutionary rate was estimated using protein identity and nonsynonymous substitution ratios (Ka)/synonymous substitution ratios (Ks) as described previously [26]. The rice genome (Osativa_323_v7.0) was downloaded from the Phytozome database v12.1 (http://phytozome.jgi.doe.gov/pz/portal.html), and orthologous gene pairs between bread wheat and rice were identified using the Blastp program (-evalue0.01; -max_target_seqs1). The protein identity between bread wheat and rice was normalized according to the length of alignment using Perl scripts. To calculate Ka, Ks, and Ka/Ks of orthologous gene pairs between bread wheat and rice, the sequences were aligned with ParaAT (-m clustalw2 -f paml); then, the program “yn00” and the “Yang and Nielsen (2000) method” of Phylogenetic Analysis by Maximum Likelihood (PAML) software were used to get the value. The identity and Ka/Ks of the *TaAGOs* were extracted from all wheat genes using Perl scripts.

### 2.3. Phylogenetic and Chromosomal Distribution Analysis of TaAGO Genes

Phylogenetic tree was constructed with MEGA 5.0 based on the maximum likelihood method using 1000 bootstraps; the most suitable amino acid substitution model “WAG” was selected based on the results of “Find Best DNA/Protein Model.” The tree was visualized using iTOL (https://itol.embl.de/). Homoeologous *TaAGOs* among the A, B, and D subgenomes were identified using phylogenetic relationships and previous classification methods [27, 28]. The chromosomal distribution of the 69 *TaAGOs* was visualized using Circos [29].

### 2.4. Expression Analysis of TaAGOs Using RNA-seq Data and Quantitative Real-Time PCR (qRT-PCR)

The expression levels of *TaAGOs* in seedlings, leaves, roots, stems, spikes, grains, stamens, and pistils were downloaded as Transcripts Per Million (TPM) from http://www.wheat-expression.com. A homoeolog expression pattern analysis for triads (a single gene copy per subgenome, i.e., A : B : D configuration of 1 : 1 : 1) with a total expression above 0.5 TPM was conducted. Homoeolog expression bias was classified as balanced (similar relative transcript abundance from A, B, and D homoeologs), A/B/D-suppressed (lower abundance of transcripts from A/B/D homoeologs), or A/B/D-dominant (higher abundance of transcripts from A/B/D homoeologs) patterns as described previously [28]. The ternary plot was generated using the R package “vcd.” Chinese Spring (*Triticum aestivum* L.) plants were grown in a growth chamber under 16 h/8 h light/dark period until anthesis stage. Flag leaves were dissected after 6 and 24 h of heat (42°C), PEG-6000 (25%), and NaCl (200 mM) treatment. Total RNA was extracted with Omega plant RNA kit (Omega Bio-tek, China). cDNA was synthesized using EasyScript (Trans). Glyceraldehyde3-phoshate dehydrogenase (GAPDH) was used as normalization control. Relative expression levels were calculated with the 2^-*ΔΔ*Ct^ method. The primer sequences used for qRT-PCR are listed in Supplementary Table 1.

## 3. Results

### 3.1. Identification of Bread Wheat AGO Genes

To identify the candidate *AGO* genes in bread wheat, we downloaded all annotated protein sequences (https://urgi.versailles.inra.fr/download/iwgsc/IWGSC_RefSeq_Annotations/v1.0/). A total number of 69 genes were identified as *TaAGO*, and the protein length ranged from 675 to 1210 amino acids (Supplementary Table 2). All of them contained N-terminal, PAZ, and PIWI domains based on the SMART analyses, suggesting that they are authentic *AGO* genes (Supplementary Figure 1).

**Figure 1:**
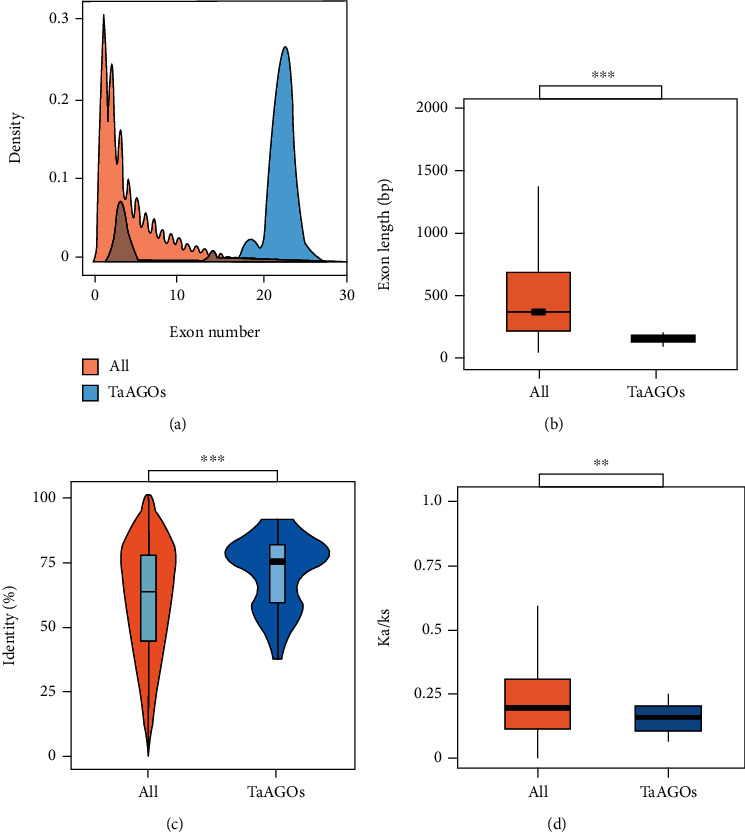
Exon structure and evolution rate analysis of *TaAGOs*. Exon number and length of *TaAGO* genes and protein identity and Ka/Ks ratio between *TaAGOs* with all the annotated bread wheat genes. (a, b) Exon number and length. (c) Protein identity. (d) Ka/Ks ratio.

We further performed exon-intron structure analyses and found that the average exon number of *TaAGOs* was 19.6, which was significantly higher than that of all the annotated wheat genes (4.6; Wilcoxon rank-sum test, *P* < 2.2*e* − 16; Figure 1(a)). Conversely, the average exon length of *TaAGOs* was dramatically lower than that of all wheat genes (275.8 bp vs. 562.6 bp; Wilcoxon rank-sum test, *P* < 2.2*e* − 16; Figure 1(b)). In addition, protein identity between *TaAGOs* and their orthologs in rice was significantly higher than that of all wheat genes (Wilcoxon rank-sum test, *P* = 8.4*e* − 6; Figure 1(c)), whereas Ka/Ks was significantly lower (Wilcoxon rank-sum test, *P* = 0.002; Figure 1(d)). All these results suggest that the evolutionary rate of *TaAGOs* is lower than that of all wheat genes. Therefore, *AGO* genes are highly conserved and their biological functions have been retained during evolutionary processes.

### 3.2. TaAGO Genes Belong to Well-Defined Subfamilies

**Figure 2:**
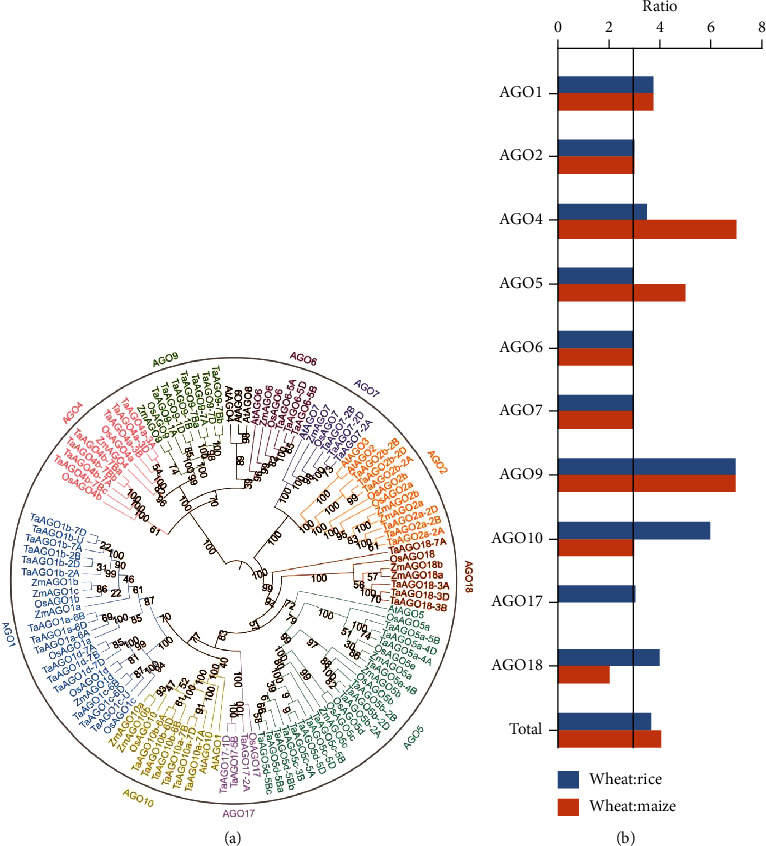
Phylogenetic relationship of AGO family genes between Arabidopsis, rice, wheat, and maize: (a) phylogenetic tree of AGO proteins; (b) the wheat : rice and wheat : maize *AGO* gene ratio in all subfamilies.

A maximum likelihood phylogenetic tree was constructed with Arabidopsis, rice, and maize AGO proteins (Figure 2(a)). TaAGOs were divided into 10 subfamilies: AGO1, AGO2, AGO4, AGO5, AGO6, AGO7, AGO9, AGO10, AGO17, and AGO18. Consistent with rice and maize AGOs, no *AGO3* and *AGO8* orthologs were detected in wheat. In some clades, gene phylogeny roughly followed species phylogeny, with one rice gene closely related to a wheat homoeolog triad (e.g., subclades AGO6 and AGO7). Duplication events made topology more complex in other subclades (e.g., subclades AGO1, AGO5, and AGO10). In addition, compared with those in rice and maize, subfamilies AGO9 were expanded even when the ploidy level was corrected (Figure 2(b)). The expansion mainly resulted from the B subgenome since the gene number in *Triticum urartu* and *Aegilops tauschii* was the same as that in maize and rice but expanded in the B subgenome of *Triticum turgidum* L. ssp. *Durum* and bread wheat (Supplementary Table 3). The expansion of AGO9 may be a result of adaptive evolution of RdDM in wheat, which helps to repress the amplified transposable element (85% in wheat vs. 35% in rice) [26, 30].

### 3.3. Chromosomal Location and High Rate of Homoeolog Retention of TaAGOs

The *TaAGO* genes were dispersed nonuniformly among the chromosomes, with chromosome 5B containing a significantly higher number of genes than expected from the chromosome length (*χ*^2^ test, *P* < 0.05; Supplementary Figure 2). This mainly resulted from segmental duplication (or whole-genome duplication) of the subfamily AGO5. Furthermore, this duplication pattern also led to more *TaAGO* genes located on chromosome 7B than expected (10.5% vs. 5.3%; Supplemental Figure 2). In addition, bread wheat contained A, B, and D subgenomes, and 41.3% of the high-confidence genes presented as triads, while 60.9% (42/69) of the *TaAGOs* presented as triads (Supplementary Table 4). The high rate of homoeolog retention indicated that the high *TaAGO* gene number mainly resulted from the segmental duplication (or whole genome duplication) in wheat.

### 3.4. Expression Pattern Analysis of TaAGOs during Wheat Development

To characterize the expression pattern of *TaAGOs* in different tissues, RNA-seq data from seedlings, roots, stems, leaves, stamens, pistils, spikes, and grains were analyzed. The results showed that 69.6% (48/69) of *TaAGOs* were expressed in at least one developmental stage (TPM > 1; Supplementary Table 5). A total of 27.5% (19/69) of genes were expressed in all the tested tissues (TPM > 1; Supplementary Table 5). Consistent with a previous study in wheat, *TaAGO4a-3A* and *TaAGO1d-7D* were expressed in both vegetative and reproductive tissues (Supplementary Table 5) [22]. In general, the expression patterns of the *TaAGOs* were comparable with the findings in rice and maize. For example, genes from subclades AGO4a, AGO5b, AGO6, and AGO9 were preferentially expressed in reproductive tissues, consistent with their roles in gametophyte development (Supplementary Table 5) [11].

**Figure 3:**
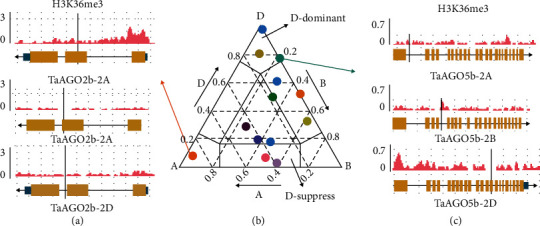
Ternary plot of the relative expression abundance of *TaAGO* triads with 1 : 1 : 1 ratio in seedling. Each circle represents the normalized expression level of A, B, and D homoeologs (b). Example of H3K36me3 regulating the homoeolog expression bias of *TaAGO2b* (a) and *TaAGO5b* (c).

Polyploidization could alter the transcriptional landscape, help to evolve new expression patterns for homoeologous gene copies, and may represent the initial steps toward sub- or neofunctionalization of wheat homoeologs [26]. To better understand the expression patterns of *TaAGOs*, we analyzed the homoeolog expression bias in different tissues. The percentage of triads displayed that similar transcript abundance from the A, B, and D homoeologs (defined as balanced) was between 16.8% and 42.9%, with an average of 33.2% (Supplementary Table 6). Further analyses indicated that histone modification may contribute to the biased expression of *TaAGOs*. For example, in seedlings, the higher level of the active marker H3K36me3 around the transcripts of *TaAGO2b-2A* than that around *TaAGO2b-2B* and *TaAGO2b-2D* may lead to an A-dominant expression pattern, while the higher levels of H3K36me3 around the transcripts of *TaAGO5b-2D* than those around *TaAGO5b-2A* and *TaAGO5b-2B* may result in a D-dominant expression pattern (Figure 3).

### 3.5. TaAGO Genes Were Influenced by Heat, Osmotic, and Salt Stress Treatments

**Figure 4:**
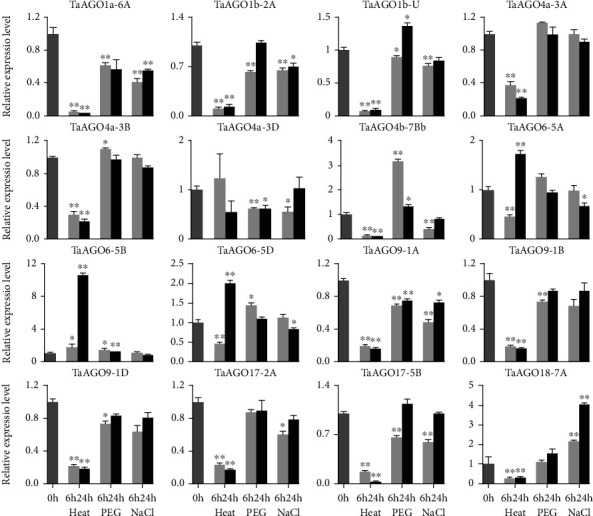
Gene expression profiles of *TaAGOs* under heat, PEG, and NaCl stress conditions. The expression level of each gene at 0 h was used as control (Student's *t*-test; ^∗^*P* < 0.05; ^∗∗^*P* < 0.01).

To understand if the expression of *TaAGOs* was affected by abiotic stresses, the transcription levels of *TaAGOs* were measured after the treatment with heat (42°C), PEG (25%), and NaCl (200 mM) for 6 and 24 hours at anthesis stage of wheat (Figure 4). The results showed that heat stress caused greater effects on the expression of *TaAGOs*. Almost all of the detected *TaAGOs*, except *TaAGO4a-3D* and members of subclade TaAGO6, were significantly suppressed by heat treatment, while 24 h treatment of heat significantly induced the expression of members of TaAGO6. In addition, the expression levels of seven *TaAGOs* were significantly affected by all the stress treatments, with a significantly downregulated expression of *TaAGO1a-6A*, *TaAGO1b-2A*, *TaAGO9-1A*, and *TaAGO17-5B*. Furthermore, *TaAGO4a* might have undergone neofunctionalization after duplication because *TaAGO4a-3A* and *TaAGO4a-3B* were significantly suppressed by heat treatments, while its homoeolog, *TaAGO4a-3D*, was not affected (Figure 4).

## 4. Discussion

RNA silencing is a conserved mechanism that regulates gene expression at the transcriptional and posttranscriptional levels. This process is mediated by several kinds of proteins including AGOs. The AGO family proteins have expanded via duplication during plant evolution, from three or fewer members in ancient unicellular green algae (e.g., *Chlamydomonas reinhardtii*) to ten or more members in flowering plants (e.g., *Arabidopsis* and rice). In this study, a total number of 69 *TaAGOs* were systematically identified and comprehensively analyzed (Supplementary Table 2). Our results indicated that segmental duplication (or whole-genome duplication) contributed to the expansion in wheat. Consistent with previous results in *Brassica* species, expansion in AGO9 clades was detected compared with that in both rice and maize (Figure 2(b)) [31]. The expansion of AGO9 presumably reflected the expansion of RdDM pathway in wheat.

In mammals, exon conservation increases with exon number and decreases with exon length [32]. In plants, *TaAGOs* have evolutionarily conserved roles. We found that *TaAGOs* showed higher exon numbers but lower exon lengths compared with those of all wheat genes (Figures 1(a) and 1(b)). Consistently, *TaAGOs* showed lower evolutionary rates, as reflected by their higher identity and lower Ka/Ks (Figures 1(c) and 1(d)). The high conservation may allow AGOs to recognize specific small RNA types in different species. For example, AGO7 binds to miR390 and promotes the production of tasiRNA in Arabidopsis, rice, and maize [7, 8, 33]. The expression pattern of many *TaAGOs* is similar to that in other plants that also indicated their conserved function among different plant species. For example, *AGO9* participated in female gamete development in both Arabidopsis and maize [11, 34]. Consistently, *TaAGO9* displayed a higher expression level in wheat pistils (Supplementary Table 5), implying that *TaAGO9* may be involved in female gamete formation. The evolutionary conservation may underline the biological importance of *AGO* gene family in wheat.

It is well known that the AGO family can protect plants from abiotic stresses. Many plant *AGO* genes showed responsive expression to a variety of abiotic stresses, including ABA, drought, high salinity, heat, and cold stresses [35, 36]. *ZmAGO18b* was significantly induced by drought, and mutation in *ZmAGO18b* led to hypersensitivity to drought stress [35]. In foxtail millet, mutation of *siAGO1b* led to decreased resistance to drought stress [37]. Chilling stress significantly upregulated the expression level of *VvAGO1*, suggesting its role in cold resistance [38]. At the transcript level, *AtAGO2* improved salt tolerance through affecting the SOS signaling cascade [39]. We observed that the expression of 12 (75%) *TaAGOs* was suppressed by heat stress. *TaAGOs* (*TaAGO6-5A* and *TaAGO6-5D*) were downregulated at 6 hours after heat stress and then significantly upregulated at 24 h, while *TaAGO6-5B* was upregulated at 6 and 24 h. Similar results were also observed in cucumber *CsAGOs*. These results indicate that *TaAGOs* may play an important role in response to high-temperature stress (Figure 4). Moreover, the expression of 12 (75.0%) and 11 (68.8%) *TaAGOs* was significantly affected by PEG and salt stress, respectively. And *TaAGOs* (*TaAGO1a-6A*, *TaAGO1b-2A*, *TaAGO1b-U*, *TaAGO4b-7Bb*, *TaAGO6-5D*, *TaAGO9-1A*, and *TaAGO17-5B*) were significantly affected by all the stress treatments, suggesting that these *TaAGOs* might participate in multiple abiotic stress responses. Collectively, our findings provide potential gene-editing targets to improve wheat performance.

Polyploidization and duplication provide additional opportunities to adapt to the environment changes through homoeologous gene expression divergence, such as neofunctionalization or subfunctionalization [28]. Bread wheat is an evolutionarily young polyploid. Its hexaploid nature provides an opportunity to study the fate of *TaAGOs* after polyploidization and analyze its contribution to the high adaptability of wheat. Based on the observation that *TaAGO4a-3A* and *TaAGO4a-3B* but not *TaAGO4a-3D* were suppressed by heat stress treatment, the evolution of *TaAGOs* could have undergone neofunctionalization (Figure 4). Furthermore, since the highly expressed homoeologs showed a higher active histone marker, H3K36me3, epigenetic modification may be associated with the expression divergence of *TaAGOs* in bread wheat (Figure 3).

Taken together, *TaAGO* genes are critical for the development and adaptability to diverse environmental conditions in bread wheat. The results of this study will broaden our understanding on the structure and function of the *AGO* gene family in wheat and help to clarify potential candidate *AGO* genes to be used for the future breeding of new wheat and other cereal crop species with increased grain yield and improved resistance to biotic and abiotic stresses.

## 5. Conclusions

The bread wheat genome possessed 69 *TaAGO* genes. We discussed the structures and performed a detailed phylogenetic analysis of this gene family. *TaAGOs* displayed slower evolutionary rate when compared with all wheat genes. Chromosome localization analysis revealed that segmental duplication (or whole-genome duplication) contributed to the expansion of the AGO gene family in wheat. Gene expression profiles revealed that *TaAGOs* were responsive to various abiotic stresses. This study provides basis for further functional characterization of these genes.

## Data Availability

The data used to support the findings of this study are included within the supplementary information file(s).

## Supplementary Materials

Supplementary Materials Supplementary Figure 1: exon-intron structure and domains of *TaAGO* genes. Supplementary Figure 2: chromosomal distribution of *TaAGOs* in wheat genomes. The *TaAGO* gene name was shown in different colors. The outer and inner track indicated the chromosome and chromosomal segment (light grey: C; grey: R2a and R2b; dark grey: R1 and R3). Connecting lines in the center of the diagram indicated the homoeologous genes. Supplementary Table 1: primers used in this study for qRT-PCR. Supplementary Table 2: list of all *TaAGO* genes identified in bread wheat. Supplementary Table 3: GeneID of subfamily AGO9 in different species. Supplementary Table 4: homoeologous groups of *TaAGO* genes. Supplementary Table 5: relative expression level (TPM) of *TaAGOs* in different tissues. Supplementary Table 6: homoeolog expression bias for triads in grain, spike, stem, leaf, root, seedling, stamen, and pistil.
