# Supplementary material for: Genome-Wide Identification and Evolutionary Analysis of Argonaute Genes in Hexaploid Bread Wheat
Source: Biomed Res Int. 2021 Jun 18;2021:9983858. doi: 10.1155/2021/9983858 (PMC8233069; doi:10.1155/2021/9983858)
Supplement: Supplementary Materials — Supplementary Figure 1: exon-intron structure and domains of TaAGO genes. Supplementary Figure 2: chromosomal distribution of TaAGOs in wheat genomes. The TaAGO gene name was shown in different colors. The outer and inner track indicated the chromosome and chromosomal segment (light grey: C; grey: R2a and R2b; dark grey: R1 and R3). Connecting lines in the center of the diagram indicated the homoeologous genes. Supplementary Table 1: primers used in this study for qRT-PCR. Supplementary Table 2: list of all TaAGO genes identified in bread wheat. Supplementary Table 3: GeneID of subfamily AGO9 in different species. Supplementary Table 4: homoeologous groups of TaAGO genes. Supplementary Table 5: relative expression level (TPM) of TaAGOs in different tissues. Supplementary Table 6: homoeolog expression bias for triads in grain, spike, stem, leaf, root, seedling, stamen, and pistil. [file 9983858.f1.zip › Supplementary Tables (1).pdf]

Supplementary Table 1 Primers used in this study for qRT-PCR

Gene Name	Forward primer (5'→3')	Reverse primer (5'→3')
GAPDH 1	TTCAACATCATTCCAAGCAGCA	CGTAACCCAAAATGCCCTTG
TaAGO1b-2A	CTATCAGGGACGTGGTGGTC	GGGCTTGATATTGGACATGC
TaAGO4a-3D	CCGTCCGTTTTTACCTTCCT	GAGAGGGCTTGGCTTTCTTT
TaAGO6-5B	ACCTGATTATGGCGGCTAAA	CCGAATCAATCCCACTAAGC
TaAGO1b-U	CTATCAGGGACGTGGTGGTC	TCGACTTGTCCACTGCTCAC
TaAGO18-7A	AGGGGTGCTACAAGGGAGTT	TATGAGAGAAAGCCCGGTTG
TaAGO6-5A	GGTCACATCTTCCAAAGCAGTTC	GGGCATGATCCAAGTCGCTTC
TaAGO6-5D	GGTGGAATGAACTCTAAGCTGGC	CCGTGATATTAGTGGCCAGCA
TaAGO4b-7Bb	ACGTGTCGAGGATTCCAACCA	CCTGGGTGGTAGAGTAGAAGCT
TaAGO4a-3A	TGCAGCAGTTGTTGGTTCTC	GGCCATCATCATCAGTTCCT
TaAGO4a-3B	TCACAGACTTATGCATCGGAGC	GCTGCAAAGCTCAACTCTACCT
TaAGO17-2A	TCTCGATTCCTAAGCGCAAT	AATGAAAACGGTCCAGATGC
TaAGO17-5B	GCTGGCACTAAGGGAGTCAG	ATGCCAAAAGATGGGCATAG
TaAGO1a-6A	GAAGCAACCAGATGCTCCTC	GGCCGTATGCTCTGGTAAAA
TaAGO9-1A	CAATACCCATTGTGGGGAAG	GAGAGGCCACTCACGAGAAC
TaAGO9-1B	GCGCTATTCTGGCAATCTTC	ACAAGCGAAGTCCTCTGCAT
TaAGO9-1D	CTCGCCAGAAAATGTTCCTC	CAAACTCCTGGAGCTCATCC

Supplementary Table 2 List of all *TaAGO* genes identified in bread wheat

Gene name	Gene ID	Chromosome	Start	End	Strand	Chromosome region	Gene length(bp)	Protein length(aa)	Subcellular localisation	Isoelectric points	Protein weight (kDa)	Meng et al. 2013	Sibisi et al. 2020
TaAGO9-1A	TraesCS1A01G445500	chr1A	593279894	593287280	+	R3	2778	925	nucl	9.26	102.45		
TaAGO9-1B	TraesCS1B01G480100	chr1B	687711448	687719941	+	R3	2784	927	nucl	9.19	102.87		
TaAGO17-1D	TraesCS1D01G151300	chr1D	209509948	209520138	-	R2b	2550	849	chlo	9.22	95.24		
TaAGO9-1D	TraesCS1D01G453600	chr1D	494465151	494473545	+	R3	2769	922	nucl	9.19	102.36		
TaAGO5b-2A	TraesCS2A01G168900	chr2A	123130416	123137441	+	R2a	3114	1037	cyto	9.69	113.02		
TaAGO17-2A	TraesCS2A01G258100	chr2A	401197692	401217644	-	R2b	2631	876	mito	9.18	98.51		
TaAGO1b-2A	TraesCS2A01G403100	chr2A	657514102	657525670	+	R2b	3363	1120	nucl	9.79	124.31		
TaAGO7-2A	TraesCS2A01G414800	chr2A	671940288	671944737	+	R3	2805	934	nucl	9.46	105.79		
TaAGO2b-2A	TraesCS2A01G419500	chr2A	675076661	675086213	-	R3	3198	1065	nucl	9.20	118.43		
TaAGO2a-2A	TraesCS2A01G419900	chr2A	675896051	675901068	-	R3	3150	1049	nucl	9.68	113.46		
TaAGO5b-2B	TraesCS2B01G195200	chr2B	172672490	172679370	+	R2a	3027	1008	cyto	9.75	109.29		
TaAGO1b-2B	TraesCS2B01G421000	chr2B	604982495	605001955	+	R2b	3309	1102	nucl	9.83	122.35		
TaAGO7-2B	TraesCS2B01G434000	chr2B	623399135	623403580	+	R2b	3153	1050	nucl	9.67	117.24		
TaAGO2b-2B	TraesCS2B01G438200	chr2B	630135789	630141803	-	R2b	3210	1069	nucl	9.16	118.34		
TaAGO2a-2B	TraesCS2B01G439000	chr2B	631202299	631207796	-	R2b	3141	1046	nucl	9.75	113.19		
TaAGO5b-2D	TraesCS2D01G176500	chr2D	119944946	119951931	+	R2a	3114	1037	cyto	9.76	112.44		
TaAGO1b-2D	TraesCS2D01G400200	chr2D	513453611	513464651	+	R2b	3300	1099	nucl	9.83	122.04		
TaAGO7-2D	TraesCS2D01G412100	chr2D	526804286	526808822	+	R3	3354	1117	chlo	9.71	124.62		
TaAGO2b-2D	TraesCS2D01G416500	chr2D	530258612	530264943	-	R3	3174	1057	nucl	9.30	117.02		
TaAGO2a-2D	TraesCS2D01G417000	chr2D	531347546	531352821	-	R3	3147	1048	nucl	9.66	113.16		
TaAGO18-3A	TraesCS3A01G116800	chr3A	86337358	86343149	+	R2a	3174	1057	nucl	9.71	115.22		

TaAGO4a-3A	TraesCS3A01G188400	chr3A	227908994	227917044	+	R2a	2751	916	nucl	9.19	101.90	*TaAGO4*	
TaAGO18-3B	TraesCS3B01G135800	chr3B	118709200	118714339	+	R2a	2526	841	cyto	9.50	94.13		
TaAGO4a-3B	TraesCS3B01G217300	chr3B	259455701	259463484	-	c	2835	944	nucl	8.78	104.74	*TaAGO4*	
TaAGO5c-3B	TraesCS3B01G287600	chr3B	461297394	461305846	+	R2b	2721	906	nucl	8.79	101.74		*TaAGO5*
TaAGO18-3D	TraesCS3D01G118600	chr3D	73377430	73383360	+	R2a	2523	840	cyto	9.38	94.13		
TaAGO4a-3D	TraesCS3D01G191600	chr3D	181110211	181118906	-	c	3009	1002	nucl	9.04	111.11	*TaAGO4*	
TaAGO5a-4A	TraesCS4A01G288300	chr4A	593278588	593285034	+	R2b	2574	857	chlo	9.38	96.40		
TaAGO5a-4B	TraesCS4B01G024000	chr4B	17229132	17237040	+	R1	3075	1024	cyto	9.57	113.00		
TaAGO5a-4D	TraesCS4D01G024100	chr4D	10684336	10690389	-	R2a	2574	857	chlo	9.39	96.62		
TaAGO6-5A	TraesCS5A01G165900	chr5A	354535082	354546071	-	R2b	2652	883	cyto	9.62	98.29		
TaAGO5c-5A	TraesCS5A01G446000	chr5A	626059348	626067611	+	R3	2550	849	nucl	9.11	96.32		
TaAGO17-5B	TraesCS5B01G116900	chr5B	203502444	203513415	+	c	2550	849	mito	9.16	95.09		
TaAGO6-5B	TraesCS5B01G162300	chr5B	299602511	299610278	+	R2b	2697	898	chlo	9.64	100.12		
TaAGO5c-5B	TraesCS5B01G451400	chr5B	622738811	622747343	-	R3	3183	1060	mito	9.56	117.21		
TaAGO5d-5Bc	TraesCS5B01G452500	chr5B	625223444	625232360	+	R3	2739	912	nucl	9.36	103.65		
TaAGO5d-5Ba	TraesCS5B01G454200	chr5B	626859479	626864234	+	R3	2028	675	nucl	9.57	76.99		
TaAGO5d-5Bb	TraesCS5B01G455700	chr5B	629970304	629985856	+	R3	2958	985	chlo	9.68	109.62		
TaAGO5a-5B	TraesCS5B01G468000	chr5B	641559960	641566692	+	R3	2622	873	chlo	9.39	98.36		
TaAGO6-5D	TraesCS5D01G169500	chr5D	265960820	265970085	+	R2b	2649	882	nucl	9.62	98.06		
TaAGO5d-5D	TraesCS5D01G192700	chr5D	296867791	296877359	-	R2b	2079	692	nucl	9.08	78.44		
TaAGO5c-5D	TraesCS5D01G454200	chr5D	501208168	501216904	-	R3	3162	1053	chlo	9.44	116.38		
TaAGO10b-6A	TraesCS6A01G178100	chr6A	199810370	199817997	-	R2a	2847	948	chlo	9.67	105.77		
TaAGO1a-6A	TraesCS6A01G254600	chr6A	471867051	471879388	-	R2b	3258	1085	nucl	9.95	120.92		
TaAGO10b-6B	TraesCS6B01G206700	chr6B	260540333	260547792	+	c	2847	948	cyto	9.77	105.97		
TaAGO1a-6B	TraesCS6B01G270900	chr6B	487774759	487789684	+	R2b	3258	1085	nucl	9.87	121.18		

TaAGO1c-6B	TraesCS6B01G466700	chr6B	716622427	716628907	-	R3	3042	1013	pero	9.97	112.03	
TaAGO10b-6D	TraesCS6D01G166300	chr6D	147875181	147882347	+	R2a	2856	951	cyto	9.64	106.26	
TaAGO1a-6D	TraesCS6D01G235900	chr6D	332541891	332556788	-	R2b	3342	1113	E.R.	9.73	124.06	
TaAGO1c-6D	TraesCS6D01G403900	chr6D	471921849	471928859	-	R3	3168	1055	chlo	10.06	116.82	
TaAGO1b-7A	TraesCS7A01G040700	chr7A	18877009	18883267	-	R1	3633	1210	vacu	9.65	133.94	
TaAGO18-7A	TraesCS7A01G067500	chr7A	33882394	33893413	+	R1	2565	854	cyto	7.99	96.16	
TaAGO10a-7A	TraesCS7A01G371200	chr7A	545059201	545065625	+	R2b	2889	962	cyto	9.65	107.52	
TaAGO4b-7A	TraesCS7A01G521000	chr7A	705135411	705141773	+	R3	2436	811	nucl	9.05	90.75	
TaAGO9-7A	TraesCS7A01G533100	chr7A	711652755	711657579	-	R3	2706	901	cyto	9.22	101.59	
TaAGO1d-7A	TraesCS7A01G557400	chr7A	729935437	729942300	+	R3	3099	1032	cyto	9.17	114.06	
TaAGO10a-7B	TraesCS7B01G256500	chr7B	475646622	475661796	+	R2b	2871	956	cyto	9.56	107.04	
TaAGO4b-7Bc	TraesCS7B01G373400	chr7B	639086798	639091157	-	R2b	2361	786	nucl	10.16	88.40	
TaAGO4b-7Ba	TraesCS7B01G437700	chr7B	704087241	704092467	+	R3	2640	879	nucl	9.06	98.74	
TaAGO4b-7Bb	TraesCS7B01G437900	chr7B	704152440	704157308	+	R3	2424	807	nucl	8.89	90.42	
TaAGO9-7Ba	TraesCS7B01G450200	chr7B	712094476	712102226	-	R3	2781	926	nucl	9.32	103.92	
TaAGO9-7Bb	TraesCS7B01G450300	chr7B	712240052	712250408	-	R3	2760	919	nucl	9.02	102.71	
TaAGO1d-7B	TraesCS7B01G482100	chr7B	739927305	739934199	+	R3	3099	1032	cyto	9.17	113.65	
TaAGO1b-7D	TraesCS7D01G036100	chr7D	18428040	18434397	-	R1	3633	1210	chlo	9.72	133.83	
TaAGO10a-7D	TraesCS7D01G351700	chr7D	452689735	452704469	+	R2b	2859	952	cyto	9.56	106.72	
TaAGO9-7D	TraesCS7D01G520400	chr7D	618038397	618043411	-	R3	2730	909	nucl	8.74	101.51	
TaAGO1d-7D	TraesCS7D01G553000	chr7D	635843887	635850770	-	R3	3096	1031	cyto	9.35	113.95	*TaAGO1b*
TaAGO1b-U	TraesCSU01G065300	chrUn	50835581	50843791	-	Na	3579	1192	nucl	9.77	131.45	
TaAGO1c-U	TraesCSU01G081900	chrUn	74810506	74817105	+	Na	2937	978	mito	9.78	108.49	

Supplementary Table 3 GeneID of subfamily AGO9 in different species

species	gene ID
Oryza sativa	LOC_Os04g06770
Zea mays	GRMZM2G141818
Triticum urartu	TuG1812G0700005670.01
Aegilops tauschii	AET1Gv21048600
Triticum turgidum L. ssp. *Durum*	TRITD1Av1G231210
	TRITD1Bv1G229290
	TRITD1Bv1G229290
Triticum aestivum	TraesCS1A01G445500
	TraesCS1B01G480100
	TraesCS1D01G453600
	TraesCS7A01G533100
	TraesCS7B01G450200
	TraesCS7B01G450300
	TraesCS7D01G520400

Supplementary Table 4 Homoeologous groups of *TaAGO* genes

**Group1**	**A:B:D=1:1:1**	
A-subgenome homoeolog	B-subgenome homoeolog	D-subgenome homoeolog
TraesCS2A01G258100	TraesCS5B01G116900	TraesCS1D01G151300
TraesCS1A01G445500	TraesCS1B01G480100	TraesCS1D01G453600
TraesCS2A01G168900	TraesCS2B01G195200	TraesCS2D01G176500
TraesCS2A01G403100	TraesCS2B01G421000	TraesCS2D01G400200
TraesCS2A01G414800	TraesCS2B01G434000	TraesCS2D01G412100
TraesCS2A01G419500	TraesCS2B01G438200	TraesCS2D01G416500
TraesCS2A01G419900	TraesCS2B01G439000	TraesCS2D01G417000
TraesCS3A01G116800	TraesCS3B01G135800	TraesCS3D01G118600
TraesCS3A01G188400	TraesCS3B01G217300	TraesCS3D01G191600
TraesCS5A01G165900	TraesCS5B01G162300	TraesCS5D01G169500
TraesCS6A01G178100	TraesCS6B01G206700	TraesCS6D01G166300
TraesCS6A01G254600	TraesCS6B01G270900	TraesCS6D01G235900
TraesCS7A01G371200	TraesCS7B01G256500	TraesCS7D01G351700
TraesCS7A01G557400	TraesCS7B01G482100	TraesCS7D01G553000

**Group2**	**A:B:D=1:3:0**		
A-subgenome homoeolog	B-subgenome homoeolog	B-subgenome homoeolog	B-subgenome homoeolog
TraesCS7A01G521000	TraesCS7B01G373400	TraesCS7B01G437700	TraesCS7B01G437900

**Group3**	**A:B:D=1:2:1**		
A-subgenome homoeolog	B-subgenome homoeolog	B-subgenome homoeolog	D-subgenome homoeolog
TraesCS4A01G288300	TraesCS4B01G024000	TraesCS5B01G468000	TraesCS4D01G024100
TraesCS5A01G446000	TraesCS3B01G287600	TraesCS5B01G451400	TraesCS5D01G454200
TraesCS7A01G533100	TraesCS7B01G450200	TraesCS7B01G450300	TraesCS7D01G520400

**Group4**	**A:B:D=1:2:1**		
B-subgenome homoeolog	B-subgenome homoeolog	B-subgenome homoeolog	D-subgenome homoeolog
TraesCS5B01G452500	TraesCS5B01G454200	TraesCS5B01G455700	TraesCS5D01G192700

**Group5**	**orphan**
TraesCS7A01G067500	

**Group6**	**other**	
TraesCS6B01G466700	TraesCS6D01G403900	TraesCSU01G081900
TraesCS7A01G040700	TraesCSU01G065300	TraesCS7D01G036100

Supplementary Table 5 Relative expression level (TPM) of *TaAGOs* in different tissues and under stress

	SRA ID	ERP016738							
**Gene name**	**Transcript**	**Seedling**	**Root**	**Stem**	**Leaf**	**Stamen**	**Pistil**	**Spike**	**Grain**
TaAGO1a-6A	TraesCS6A01G254600	2.87	12.81	4.81	2.46	0.81	0.30	0.65	0.10
TaAGO1a-6B	TraesCS6B01G270900	3.79	12.38	7.72	4.91	1.78	0.56	1.93	0.31
TaAGO1a-6D	TraesCS6D01G235900	1.43	2.77	5.83	0.07	0.15	0.16	4.22	0.00
TaAGO1b-2A	TraesCS2A01G403100	0.04	0.08	0.00	0.10	0.06	0.00	0.00	0.01
TaAGO1b-2B	TraesCS2B01G421000	0.12	0.00	0.09	0.02	0.00	0.08	0.06	0.01
TaAGO1b-2D	TraesCS2D01G400200	8.95	12.23	8.76	7.64	5.17	13.18	20.71	1.36
TaAGO1b-7A	TraesCS7A01G040700	12.83	17.57	8.71	10.81	5.44	13.15	24.00	43.20
TaAGO1b-7D	TraesCS7D01G036100	4.86	13.06	5.35	6.98	3.71	10.12	9.66	22.19
TaAGO1b-U	TraesCSU01G065300	9.38	19.92	9.36	11.08	6.20	17.18	25.22	20.07
TaAGO1c-6B	TraesCS6B01G466700	12.61	5.48	6.36	10.86	7.86	12.10	17.99	2.75
TaAGO1c-6D	TraesCS6D01G403900	6.69	6.44	9.07	5.20	7.91	9.90	17.10	3.59
TaAGO1c-U	TraesCSU01G081900	9.42	5.43	7.75	9.41	4.24	5.88	13.81	4.51
TaAGO1d-7A	TraesCS7A01G557400	0.05	0.00	0.00	0.04	0.25	1.42	11.61	0.01
TaAGO1d-7B	TraesCS7B01G482100	0.15	0.00	1.21	0.38	1.59	23.53	13.11	0.01
TaAGO1d-7D	TraesCS7D01G553000	0.31	0.05	0.01	0.32	1.09	2.50	14.76	0.00
TaAGO2a-2A	TraesCS2A01G419900	0.00	3.61	0.21	0.40	3.83	7.16	0.99	0.14
TaAGO2a-2B	TraesCS2B01G439000	5.06	17.09	6.70	6.95	4.71	9.30	10.78	3.38
TaAGO2a-2D	TraesCS2D01G417000	5.45	25.06	12.82	5.56	7.08	10.90	21.26	6.99
TaAGO2b-2A	TraesCS2A01G419500	0.52	0.11	0.03	0.80	0.95	0.32	0.71	0.04
TaAGO2b-2B	TraesCS2B01G438200	0.02	0.05	0.00	0.02	0.20	0.10	0.08	0.01
TaAGO2b-2D	TraesCS2D01G416500	0.05	0.02	0.00	0.00	0.75	0.19	0.19	0.02
TaAGO4a-3A	TraesCS3A01G188400	31.15	47.77	39.84	26.94	34.10	223.39	126.76	30.29
TaAGO4a-3B	TraesCS3B01G217300	34.51	63.77	60.73	32.97	47.02	181.29	154.65	17.97
TaAGO4a-3D	TraesCS3D01G191600	4.35	6.12	2.16	2.08	10.06	72.91	18.34	6.06
TaAGO4b-7A	TraesCS7A01G521000	0.20	0.91	0.52	0.58	0.11	0.32	0.29	0.44
TaAGO4b-7Ba	TraesCS7B01G437700	0.00	0.00	0.00	0.00	0.02	0.02	0.01	0.00
TaAGO4b-7Bb	TraesCS7B01G437900	0.00	0.00	0.00	0.00	0.07	0.00	0.00	0.00
TaAGO4b-7Bc	TraesCS7B01G373400	0.02	0.00	0.22	0.09	0.04	0.03	0.37	0.00
TaAGO5a-4A	TraesCS4A01G288300	0.00	0.00	0.00	0.00	0.03	0.00	0.05	0.00
TaAGO5a-4B	TraesCS4B01G024000	0.00	0.00	0.00	0.00	0.35	0.00	0.02	0.43
TaAGO5a-4D	TraesCS4D01G024100	0.00	0.00	0.00	0.03	0.04	0.00	0.17	0.00
TaAGO5a-5B	TraesCS5B01G468000	0.09	0.03	1.09	0.09	0.37	0.08	1.34	0.01
TaAGO5b-2A	TraesCS2A01G168900	0.00	0.51	0.37	0.00	3.79	67.76	17.52	13.29
TaAGO5b-2B	TraesCS2B01G195200	0.29	0.71	0.75	0.52	2.65	30.89	9.41	5.55
TaAGO5b-2D	TraesCS2D01G176500	0.99	1.57	2.70	1.88	7.80	68.27	21.02	12.83
TaAGO5c-3B	TraesCS3B01G287600	1.36	0.87	3.02	0.66	0.36	0.08	2.91	0.13
TaAGO5c-5A	TraesCS5A01G446000	0.43	0.00	0.00	0.00	0.15	0.21	0.00	0.52
TaAGO5c-5B	TraesCS5B01G451400	0.14	0.00	0.00	0.00	0.11	0.00	0.00	0.00
TaAGO5c-5D	TraesCS5D01G454200	0.25	0.14	0.40	0.21	0.20	0.08	0.87	0.16

TaAGO5d-5Bc	TraesCS5B01G452500	0.02	0.12	0.03	0.01	0.09	0.08	0.10	0.00
TaAGO5d-5Ba	TraesCS5B01G454200	1.68	2.23	1.39	1.60	0.72	0.99	7.10	0.62
TaAGO5d-5Bb	TraesCS5B01G455700	0.47	0.33	0.50	0.48	0.25	0.22	1.38	0.11
TaAGO5d-5D	TraesCS5D01G192700	0.53	0.31	0.97	0.34	0.85	1.13	2.85	0.35
TaAGO6-5A	TraesCS5A01G165900	5.15	10.48	0.76	1.94	2.05	16.52	8.48	2.47
TaAGO6-5B	TraesCS5B01G162300	2.96	7.92	1.84	2.01	1.22	7.64	6.03	1.53
TaAGO6-5D	TraesCS5D01G169500	3.27	7.96	1.10	1.89	3.25	11.34	6.89	1.66
TaAGO7-2A	TraesCS2A01G414800	0.44	0.87	0.75	0.44	0.06	1.12	0.99	0.02
TaAGO7-2B	TraesCS2B01G434000	0.39	0.60	0.60	0.76	0.06	1.03	5.29	0.04
TaAGO7-2D	TraesCS2D01G412100	0.20	0.52	0.06	0.18	0.08	1.36	1.01	0.00
TaAGO9-1A	TraesCS1A01G445500	9.98	11.65	29.63	7.93	7.42	44.55	22.01	13.74
TaAGO9-1B	TraesCS1B01G480100	17.73	17.00	1.85	7.67	10.79	40.57	27.67	8.43
TaAGO9-1D	TraesCS1D01G453600	27.47	19.01	9.43	17.81	17.57	70.88	39.60	15.03
TaAGO9-7A	TraesCS7A01G533100	0.00	0.00	0.00	0.00	0.15	0.00	0.00	0.00
TaAGO9-7Ba	TraesCS7B01G450200	0.00	0.00	0.49	0.00	0.00	0.68	0.40	0.03
TaAGO9-7Bb	TraesCS7B01G450300	0.00	0.00	0.00	0.00	0.00	0.00	0.00	0.00
TaAGO9-7D	TraesCS7D01G520400	0.00	0.00	0.00	0.00	0.00	0.01	0.00	0.00
TaAGO10a-7A	TraesCS7A01G371200	0.46	0.45	0.10	0.05	0.57	0.36	1.09	0.00
TaAGO10a-7B	TraesCS7B01G256500	5.04	23.14	0.89	3.82	3.01	2.99	4.21	0.19
TaAGO10a-7D	TraesCS7D01G351700	2.64	7.29	0.37	0.94	1.40	1.37	2.52	0.00
TaAGO10b-6A	TraesCS6A01G178100	0.15	0.15	0.00	0.00	0.21	0.00	0.19	0.00
TaAGO10b-6B	TraesCS6B01G206700	0.11	1.01	0.00	0.00	0.03	0.02	0.40	0.09
TaAGO10b-6D	TraesCS6D01G166300	1.11	3.13	0.04	0.44	0.49	0.46	0.91	0.17
TaAGO17-1D	TraesCS1D01G151300	0.50	2.81	1.61	0.00	0.51	0.52	2.63	0.24
TaAGO17-2A	TraesCS2A01G258100	7.20	12.15	15.71	8.03	4.56	4.58	13.62	2.70
TaAGO17-5B	TraesCS5B01G116900	10.68	13.81	24.28	13.02	4.95	6.30	13.39	6.28
TaAGO18-3A	TraesCS3A01G116800	0.02	0.07	1.86	1.54	8.73	0.10	1.20	0.08
TaAGO18-3B	TraesCS3B01G135800	0.05	0.16	0.41	0.70	10.20	0.05	1.43	0.00
TaAGO18-3D	TraesCS3D01G118600	0.01	0.16	3.58	1.23	11.37	0.06	1.23	0.00
TaAGO18-7A	TraesCS7A01G067500	0.01	0.01	1.42	0.11	0.00	0.24	0.43	0.00

Supplementary Table 6 Homoeolog expression bias for triads in grain, spike, stem, leaf, root, seedling, stamen and pistil

Triad	Gene name	Gene ID	Grain		Spike		Stem		Leaf		Root		Seedling		Stamen		Pistil	
			TPM	Category	TPM	Category	TPM	Category	TPM	Category	TPM	Category	TPM	Category	TPM	Category	TPM	Category
	TaAGO17-2A	TraesCS2A01G258100	2.70	D suppressed	13.62	D suppressed	15.71	D suppressed	8.03	D suppressed	12.15	D suppressed	7.20	D suppressed	4.56	D suppressed	4.58	D suppressed
Triad 1	TaAGO17-5B	TraesCS5B01G116900	6.28	D suppressed	13.39	D suppressed	24.28	D suppressed	13.02	D suppressed	13.81	D suppressed	10.68	D suppressed	4.95	D suppressed	6.30	D suppressed
	TaAGO17-1D	TraesCS1D01G151300	0.24	D suppressed	2.63	D suppressed	1.61	D suppressed	0.00	D suppressed	2.81	D suppressed	0.50	D suppressed	0.51	D suppressed	0.52	D suppressed
	TaAGO9-1A	TraesCS1A01G445500	13.74	balanced	22.01	balanced	29.63	B suppressed	7.93	balanced	11.65	balanced	9.98	balanced	7.42	balanced	44.55	balanced
Triad 2	TaAGO9-1B	TraesCS1B01G480100	8.43	balanced	27.67	balanced	1.85	B suppressed	7.67	balanced	17.00	balanced	17.73	balanced	10.79	balanced	40.57	balanced
	TaAGO9-1D	TraesCS1D01G453600	15.03	balanced	39.60	balanced	9.43	B suppressed	17.81	balanced	19.01	balanced	27.47	balanced	17.57	balanced	70.88	balanced
	TaAGO5b-2A	TraesCS2A01G168900	13.29	balanced	17.52	balanced	0.37	D dominant	0.00	D dominant	0.51	balanced	0.00	D dominant	3.79	balanced	67.76	balanced
Triad 3	TaAGO5b-2B	TraesCS2B01G195200	5.55	balanced	9.41	balanced	0.75	D dominant	0.52	D dominant	0.71	balanced	0.29	D dominant	2.65	balanced	30.89	balanced
	TaAGO5b-2D	TraesCS2D01G176500	12.83	balanced	21.02	balanced	2.70	D dominant	1.88	D dominant	1.57	balanced	0.99	D dominant	7.80	balanced	68.27	balanced
	TaAGO1b-2A	TraesCS2A01G403100	0.01	D dominant	0.00	D dominant	0.00	D dominant	0.10	D dominant	0.08	D dominant	0.04	D dominant	0.06	D dominant	0.00	D dominant
Triad 4	TaAGO1b-2B	TraesCS2B01G421000	0.01	D dominant	0.06	D dominant	0.09	D dominant	0.02	D dominant	0.00	D dominant	0.12	D dominant	0.00	D dominant	0.08	D dominant
	TaAGO1b-2D	TraesCS2D01G400200	1.36	D dominant	20.71	D dominant	8.76	D dominant	7.64	D dominant	12.23	D dominant	8.95	D dominant	5.17	D dominant	13.18	D dominant
	TaAGO7-2A	TraesCS2A01G414800	0.02	not expressed	0.99	B dominant	0.75	D suppressed	0.44	D suppressed	0.87	balanced	0.44	balanced	0.06	not expressed	1.12	balanced
Triad 5	TaAGO7-2B	TraesCS2B01G434000	0.04	not expressed	5.29	B dominant	0.60	D suppressed	0.76	D suppressed	0.60	balanced	0.39	balanced	0.06	not expressed	1.03	balanced
	TaAGO7-2D	TraesCS2D01G412100	0.00	not expressed	1.01	B dominant	0.06	D suppressed	0.18	D suppressed	0.52	balanced	0.20	balanced	0.08	not expressed	1.36	balanced
	TaAGO2b-2A	TraesCS2A01G419500	0.04	not expressed	0.71	A dominant	0.03	not expressed	0.80	A dominant	0.11	not expressed	0.52	A dominant	0.95	B suppressed	0.32	B suppressed
Triad 6	TaAGO2b-2B	TraesCS2B01G438200	0.01	not expressed	0.08	A dominant	0.00	not expressed	0.02	A dominant	0.05	not expressed	0.02	A dominant	0.20	B suppressed	0.10	B suppressed
	TaAGO2b-2D	TraesCS2D01G416500	0.02	not expressed	0.19	A dominant	0.00	not expressed	0.00	A dominant	0.02	not expressed	0.05	A dominant	0.75	B suppressed	0.19	B suppressed
Triad 7	TaAGO2a-2A	TraesCS2A01G419900	0.14	A suppressed	0.99	A suppressed	0.21	A suppressed	0.40	A suppressed	3.61	A suppressed	0.00	A suppressed	3.83	balanced	7.16	balanced

	TaAGO2a-2B	TraesCS2B01G439000	3.38	A suppressed	10.78	A suppressed	6.70	A suppressed	6.95	A suppressed	17.09	A suppressed	5.06	A suppressed	4.71	balanced	9.30	balanced
	TaAGO2a-2D	TraesCS2D01G417000	6.99	A suppressed	21.26	A suppressed	12.82	A suppressed	5.56	A suppressed	25.06	A suppressed	5.45	A suppressed	7.08	balanced	10.90	balanced
	TaAGO18-3A	TraesCS3A01G116800	0.08	not expressed	1.20	balanced	1.86	B suppressed	1.54	balanced	0.07	not expressed	0.02	not expressed	8.73	balanced	0.10	not expressed
Triad 8	TaAGO18-3B	TraesCS3B01G135800	0.00	not expressed	1.43	balanced	0.41	B suppressed	0.70	balanced	0.16	not expressed	0.05	not expressed	10.20	balanced	0.05	not expressed
	TaAGO18-3D	TraesCS3D01G118600	0.00	not expressed	1.23	balanced	3.58	B suppressed	1.23	balanced	0.16	not expressed	0.01	not expressed	11.37	balanced	0.06	not expressed
	TaAGO4a-3A	TraesCS3A01G188400	30.29	D suppressed	126.76	D suppressed	39.84	D suppressed	26.94	D suppressed	47.77	D suppressed	31.15	D suppressed	34.10	D suppressed	223.39	D suppressed
Triad 9	TaAGO4a-3B	TraesCS3B01G217300	17.97	D suppressed	154.65	D suppressed	60.73	D suppressed	32.97	D suppressed	63.77	D suppressed	34.51	D suppressed	47.02	D suppressed	181.29	D suppressed
	TaAGO4a-3D	TraesCS3D01G191600	6.06	D suppressed	18.34	D suppressed	2.16	D suppressed	2.08	D suppressed	6.12	D suppressed	4.35	D suppressed	10.06	D suppressed	72.91	D suppressed
	TaAGO6-5A	TraesCS5A01G165900	2.47	balanced	8.48	balanced	0.76	balanced	1.94	balanced	10.48	balanced	5.15	balanced	2.05	balanced	16.52	balanced
Triad 10	TaAGO6-5B	TraesCS5B01G162300	1.53	balanced	6.03	balanced	1.84	balanced	2.01	balanced	7.92	balanced	2.96	balanced	1.22	balanced	7.64	balanced
	TaAGO6-5D	TraesCS5D01G169500	1.66	balanced	6.89	balanced	1.10	balanced	1.89	balanced	7.96	balanced	3.27	balanced	3.25	balanced	11.34	balanced
	TaAGO10b-6A	TraesCS6A01G178100	0.00	not expressed	0.19	A suppressed	0.00	not expressed	0.00	not expressed	0.15	A suppressed	0.15	D dominant	0.21	B suppressed	0.00	not expressed
Triad 11	TaAGO10b-6B	TraesCS6B01G206700	0.09	not expressed	0.40	A suppressed	0.00	not expressed	0.00	not expressed	1.01	A suppressed	0.11	D dominant	0.03	B suppressed	0.02	not expressed
	TaAGO10b-6D	TraesCS6D01G166300	0.17	not expressed	0.91	A suppressed	0.04	not expressed	0.44	not expressed	3.13	A suppressed	1.11	D dominant	0.49	B suppressed	0.46	not expressed
	TaAGO1a-6A	TraesCS6A01G254600	0.10	not expressed	0.65	A suppressed	4.81	balanced	2.46	D suppressed	12.81	D suppressed	2.87	balanced	0.81	D suppressed	0.30	D suppressed
Triad 12	TaAGO1a-6B	TraesCS6B01G270900	0.31	not expressed	1.93	A suppressed	7.72	balanced	4.91	D suppressed	12.38	D suppressed	3.79	balanced	1.78	D suppressed	0.56	D suppressed
	TaAGO1a-6D	TraesCS6D01G235900	0.00	not expressed	4.22	A suppressed	5.83	balanced	0.07	D suppressed	2.77	D suppressed	1.43	balanced	0.15	D suppressed	0.16	D suppressed
	TaAGO10a-7A	TraesCS7A01G371200	0.00	not expressed	1.09	A suppressed	0.10	A suppressed	0.05	B dominant	0.45	B dominant	0.46	A suppressed	0.57	A suppressed	0.36	A suppressed
Triad 13	TaAGO10a-7B	TraesCS7B01G256500	0.19	not expressed	4.21	A suppressed	0.89	A suppressed	3.82	B dominant	23.14	B dominant	5.04	A suppressed	3.01	A suppressed	2.99	A suppressed
	TaAGO10a-7D	TraesCS7D01G351700	0.00	not expressed	2.52	A suppressed	0.37	A suppressed	0.94	B dominant	7.29	B dominant	2.64	A suppressed	1.40	A suppressed	1.37	A suppressed
	TaAGO1d-7A	TraesCS7A01G557400	0.01	not expressed	11.61	balanced	0.00	B dominant	0.04	A suppressed	0.00	not expressed	0.05	A suppressed	0.25	A suppressed	1.42	B dominant
Triad 14	TaAGO1d-7B	TraesCS7B01G482100	0.01	not expressed	13.11	balanced	1.21	B dominant	0.38	A suppressed	0.00	not expressed	0.15	A suppressed	1.59	A suppressed	23.53	B dominant
	TaAGO1d-7D	TraesCS7D01G553000	0.00	not expressed	14.76	balanced	0.01	B dominant	0.32	A suppressed	0.05	not expressed	0.31	A suppressed	1.09	A suppressed	2.50	B dominant
