# Supplementary figures and images for: Genome-Wide Identification and Evolutionary Analysis of Argonaute Genes in Hexaploid Bread Wheat
Source: Biomed Res Int. 2021 Jun 18;2021:9983858. doi: 10.1155/2021/9983858 (PMC8233069; doi:10.1155/2021/9983858)

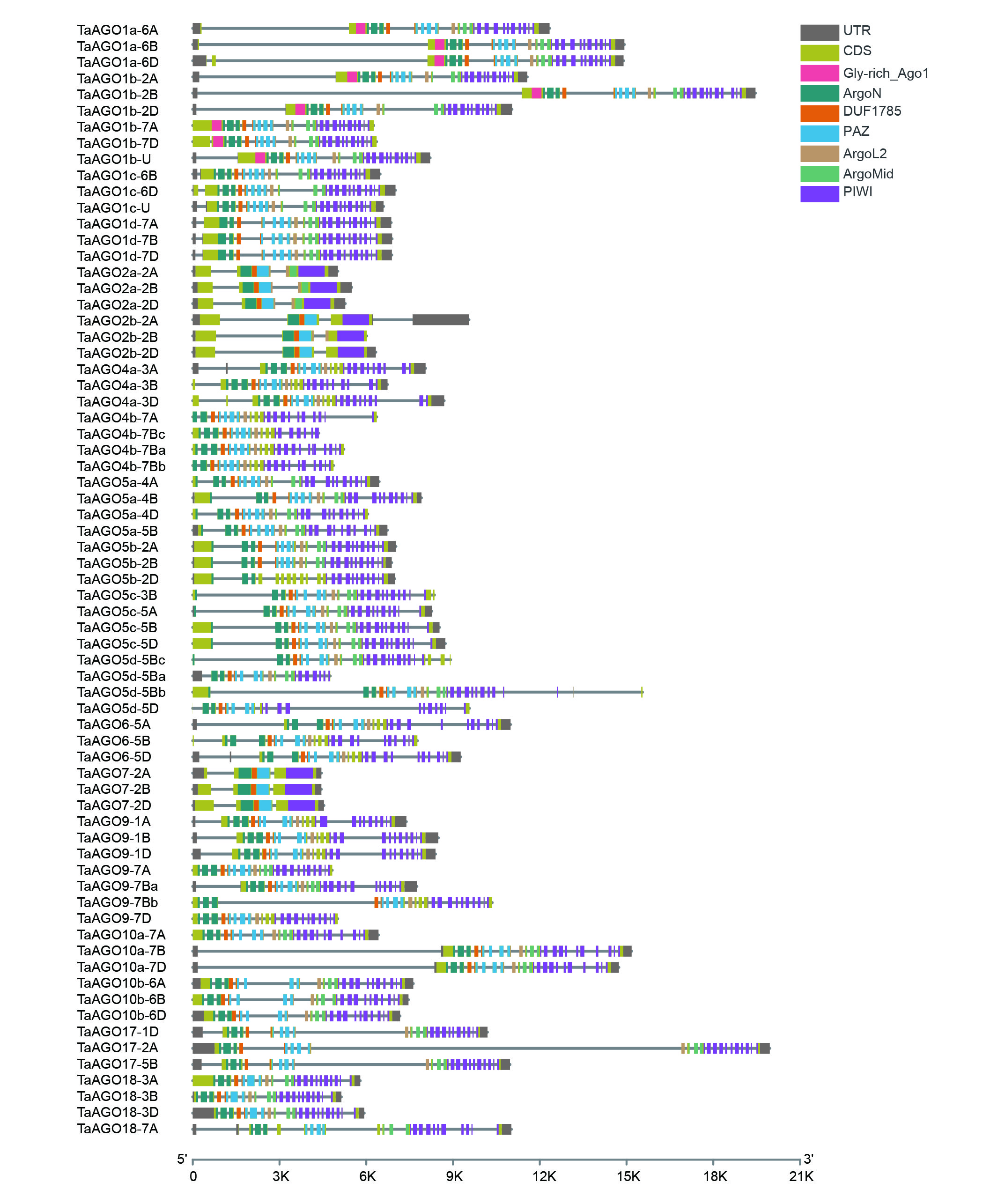

Supplement: Supplementary Materials — Supplementary Figure 1: exon-intron structure and domains of TaAGO genes. Supplementary Figure 2: chromosomal distribution of TaAGOs in wheat genomes. The TaAGO gene name was shown in different colors. The outer and inner track indicated the chromosome and chromosomal segment (light grey: C; grey: R2a and R2b; dark grey: R1 and R3). Connecting lines in the center of the diagram indicated the homoeologous genes. Supplementary Table 1: primers used in this study for qRT-PCR. Supplementary Table 2: list of all TaAGO genes identified in bread wheat. Supplementary Table 3: GeneID of subfamily AGO9 in different species. Supplementary Table 4: homoeologous groups of TaAGO genes. Supplementary Table 5: relative expression level (TPM) of TaAGOs in different tissues. Supplementary Table 6: homoeolog expression bias for triads in grain, spike, stem, leaf, root, seedling, stamen, and pistil. [file 9983858.f1.zip › Supplementary Figure 1.jpg]

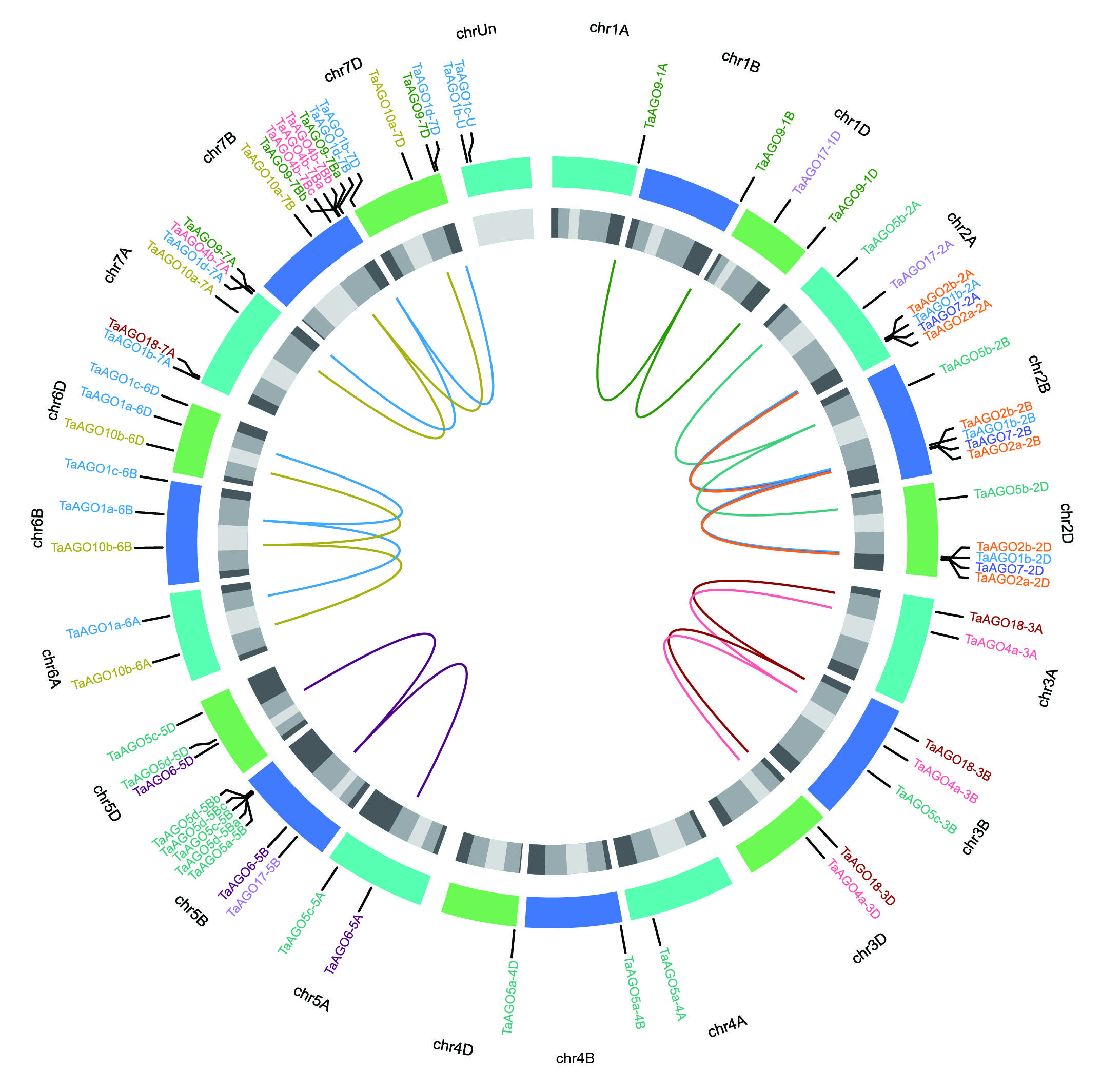

Supplement: Supplementary Materials — Supplementary Figure 1: exon-intron structure and domains of TaAGO genes. Supplementary Figure 2: chromosomal distribution of TaAGOs in wheat genomes. The TaAGO gene name was shown in different colors. The outer and inner track indicated the chromosome and chromosomal segment (light grey: C; grey: R2a and R2b; dark grey: R1 and R3). Connecting lines in the center of the diagram indicated the homoeologous genes. Supplementary Table 1: primers used in this study for qRT-PCR. Supplementary Table 2: list of all TaAGO genes identified in bread wheat. Supplementary Table 3: GeneID of subfamily AGO9 in different species. Supplementary Table 4: homoeologous groups of TaAGO genes. Supplementary Table 5: relative expression level (TPM) of TaAGOs in different tissues. Supplementary Table 6: homoeolog expression bias for triads in grain, spike, stem, leaf, root, seedling, stamen, and pistil. [file 9983858.f1.zip › Supplementary Figure 2.jpg]
